# Beverages – a scoping review for Nordic Nutrition Recommendations 2023

**DOI:** 10.29219/fnr.v68.10458

**Published:** 2024-04-02

**Authors:** Emily Sonestedt, Marko Lukic

**Affiliations:** 1 Department of Clinical Sciences Malmö, Lund University, Malmö, Sweden; 2 Department of Community Medicine, Faculty of Health Sciences, UiT – The Arctic University of Norway, Tromsø, Norway

**Keywords:** beverages, coffee, tea, sugar-sweetened beverages, sweeteners, dietary guidelines

## Abstract

***Background:*** Coffee, tea, sugar-sweetened beverages (SSBs), and low- and no-calorie sweetened beverages (LNCSBs) are generally frequently consumed in the Nordic and Baltic countries. These beverages have also been related to potential health effects. This scoping review describes the evidence for the role of coffee, tea, SSBs, and LNCSBs for health-related outcomes as a basis for setting and updating food-based dietary guidelines. We used evidence from several qualified systematic reviews (i.e. World Cancer Research Fund, US Dietary Guidelines Advisory Committee, European Food Safety Authority, and World Health Organization) and performed a search for additional systematic reviews. The evidence suggests that moderate coffee and tea consumption do not have long-term adverse health effects. The long-term favorable effects of coffee consumption are related to reduced risk of endometrial and liver cancer, type 2 diabetes, and cardiovascular deaths. However, results from randomized controlled trials (RCTs) suggest that coffee brews that are rich in diterpenes, such as boiled coffee, increase serum cholesterol concentrations. High caffeine intake in pregnancy is associated with higher risk of pregnancy loss, preterm birth, and low birth weight. High consumption of SSBs has been associated with increased risk of obesity, type 2 diabetes, hypertension, and cardiovascular disease, based on data from RCTs and prospective cohort studies. The consumption of LNCSBs may result in a small reduction in body weight in adults, likely mediated through the effect of reduced energy intake, but has neutral effects on other cardiometabolic risk markers using evidence from RCTs. However, evidence from observational studies indicates increased risk of cardiometabolic diseases among high LNCSB consumers. In conclusion, current evidence suggests that moderate coffee and tea consumption have no long-term adverse health effects. The evidence of beneficial effects of coffee consumption on liver and endometrial cancer risk, and some cardiovascular outcomes, comes from observational studies. High consumption of boiled coffee should be avoided due to negative effect on lipid profile. Pregnant women should not exceed the recommended daily dose of caffeine intake of 200 mg set by the European Food Safety Authority as a safe level for the fetus. High consumption of SSBs has consistently been associated with adverse health effects, which is mainly due to excess energy intake, and should be limited. The conflicting results from RCTs and observational studies regarding LNCSBs may be due to revere causation and should be explored further.

## Popular scientific summary

- A moderate consumption of coffee or tea may have beneficial health effects such as reduced risk of liver cancer and cardiovascular disease.
- The consumption of unfiltered/boiled coffee can increase serum cholesterol levels.
- Pregnant and lactating women should not exceed the recommended daily dose of caffeine.
- Sugar-sweetened beverages are associated with increased risk of obesity, type 2 diabetes, hypertension, and cardiovascular disease.
- Replacing sugar-sweetened beverages with low- or no-calorie-sweetened beverages may result in a small reduction in body weight.

Beverages can be broadly defined as any type of drink. They can provide energy and nutrients or be free from both energy and nutrients. This scoping review covers coffee, tea, sugar-sweetened beverages (SSBs), and low- and no-calorie sweetened beverages (LNCSBs).

Coffee is a hot beverage made from roasted and ground coffee beans. It is mostly made either of *Coffea arabica* or *Coffea canephora* (s. *Coffea robusta*), two of the species of the genus of plants known as *Coffea* (1). The main types of coffee beverages that are consumed in the Nordic countries are filtered, instant, boiled, and different types of espresso-like coffee beverages. Coffee consists of numerous components whose concentrations and bioavailability depend on coffee type, roasting, and brewing methods. Caffeine (1,3,7-trimethylxanthine) is an alkaloid, and the content in coffee beverage is dependent on brewing method. On average, one cup of espresso coffee (60 mL) contains around 80 mg of caffeine, and one cup of filtered coffee (200 mL) contains around 90 mg of caffeine (2). Compared to filtered coffee, boiled coffee has on average higher concentration of caffeine, with one study reporting that the average difference was around 30 mg per 100 mL (3–5). Caffeine has an average half-life of 5 h in plasma in healthy individuals. Nonetheless, the duration for the half-life elimination can vary from 1.5 to 9.5 h (2). There is also coffee without any caffeine, that is, decaffeinated coffee. Other substances in coffee with potential effects are coffee diterpenes and chlorogenic acids (6). Tea is a beverage made by infusing dried leaves of the tea plant *Camelia sinensis* in hot water. There are many different types of tea, including black tea, green tea, white tea, and herbal tea. The concentration of caffeine in tea ranges from 14 to 61 mg per cup, depending on the type of tea (7). One cup of black tea (220 mL) contains around 50 mg of caffeine (2).

SSBs are often defined as soft drinks, fruit drinks, and sports and energy drinks with added sugar. Flavored milk or coffee and tea drinks with added sugar are not included in this definition. Sucrose, which consists of one molecule of glucose and one molecule of fructose, is the most commonly used sweetener in the Nordic and Baltic countries. In other countries, for example, the United States, high-fructose corn syrups (usually consisting of 45% glucose and 55% fructose) are also commonly used as sweetener. LNCSBs are soft drinks and sport and energy drinks, where sweeteners with no or with very low-calorie content have been used. Because these sweeteners have a much higher sweetness, very small amounts are required of these substances for the same sweetness. The sweeteners can be artificially or not. The most common sweeteners in the Nordic and Baltic countries are aspartame, acesulfame potassium, and sucralose that are derived from chemical sources, and steviol glucosides that are derived from natural sources. These substances are 120 to 750 times sweeter than sucrose (8). All approved sweeteners have been examined very carefully and have been assessed as safe to be used as food additives by the European Food Safety Authority (EFSA) (9). However, there are regulations for how they may be used, that is, in which types of food and in what quantity. The acceptable daily intake (ADI) for aspartame is 40 mg per kg body weight. EFSA has estimated that high consumers (95th percentile) in Europe have an intake of 5.5 milligrams per kg body weight (9).

In the Nordic Nutrition Recommendations (NNR) 2012, the recommendation was to limit the intake of added sugars below 10 percentage of energy intake for adults and children above 6 months of age (10). The recommendation has, therefore, been to limit the intake of SSBs and sugar-rich foods in several of the Nordic countries (11–13). There has not been any food-based dietary guidelines on coffee, tea, or LNCSBs in any of the Nordic or Baltic countries. The EFSA recommends that single doses of caffeine up to 200 mg and the intake up to 400 mg per day from all sources do not raise safety concerns for the general healthy adult population. For children, the current recommendation on a safe level of caffeine intake is 3 mg per kilogram of body weight per day. For pregnant and lactating women, the recommendation for caffeine intake is set to maximum 200 mg per day (2).

Coffee, tea, SSBs, and LNCSBs are generally frequently consumed in the Nordic and Baltic population. In addition, because of current focus on reducing intake of added sugar, low-calorie sweeteners are more frequently added to foods and beverages as a replacement for sugar. The intake of LNCSBs has therefore increased during the last decade (8). The aim of this scoping review is to describe the totality of evidence for the role of coffee, tea, SSBs, and LNCSB, for health-related outcomes as a basis for setting and updating food-based dietary guidelines (Box 1).

*Box 1.* Background papers for Nordic Nutrition Recommendations 2023

- This paper is one of many scoping reviews commissioned as part of the Nordic Nutrition Recommendations 2023 (NNR2023) project (14)
- The papers are included in the extended NNR2023 report but, for transparency, these scoping reviews are also published in Food & Nutrition Research
- The scoping reviews have been peer reviewed by independent experts in the research field according to the standard procedures of the journal
- The scoping reviews have also been subjected to public consultations (see report to be published by the NNR2023 project)
- The NNR2023 committee has served as the editorial board
- While these papers are a main fundament, the NNR2023 committee has the sole responsibility for setting dietary reference values in the NNR2023 project

## Methods

The review follows the protocol developed within the NNR2023 project (14). The sources of evidence used in the review follow the eligibility criteria described previously (15). No *de novo* systematic reviews were available for inclusion as a source of evidence in this review (16). We used qualified systematic reviews from the World Cancer Research Fund (WCRF) for the effect of coffee and tea on cancer risk (17). For the effect of SSB on various health outcomes, information from the following qualified systematic reviews was used: The EFSA Scientific opinion on upper level for sugar 2022 (18) and a systematic review for the US Dietary Guidelines 2020 (19). For LNCSBs, we used a systematic review for the US Dietary Guidelines 2020 (19) and from the World Health Organization (WHO) on health effects of the use of non-sugar sweeteners (20). In addition, we performed literature search for systematic reviews on the effect of coffee, tea, and LNCSBs on various health outcomes. The search on coffee and tea was conducted in PubMed using the following search string: coffee OR caffeine OR tea AND systematic review. The search resulted in 132 articles, of which 103 were read in full text and 47 articles were included in this review as sources of evidence. The search on LNCSBs was conducted in PubMed on November 19th, 2021 using the following search string: artificially sweetened beverages OR low-calorie sweetened AND systematic review. The search resulted in 34 articles, of which 21 were read in full text and 13 articles were included in this review as sources of evidence. A separate search was conducted in December 2022 in PubMed on Mendelian randomization studies that are using genetic variants as proxy for coffee consumption. The following search terms were used: Mendelian randomization AND coffee. The search resulted in 98 articles (original studies), of which 29 had relevant outcomes and described the result with enough details (22 were published between 2020 and 2022).

## Diet intake in the Nordic and Baltic countries

The intake of coffee, tea, and soft drinks from the most recent national dietary surveys in adults (2010–2020) is shown in Table 1. The mean intake of coffee ranges from 252 g/day among women in Estonia to 706 g/day among men in Denmark. Mean intake of tea ranges from 40 g/day among men in Iceland to 240 g/day among women in Estonia. Mean intake of soft drinks, cordials, etc. ranges from 36 g/day among women in Lithuania to 282 g/day among men in Norway (21). Data on the intake of LNCSB separately were not available; however, the data from sales statistics indicate a rapid increase of LNCSB in the last decade in Nordic countries (22).

**Table 1:** Consumption of soft drinks, coffee, and tea in the Nordic and Baltic countries

Country, year (age group)	Gender	Soft drinks, cordials, etc.	Coffee	Tea
Denmark, 2011 (18–75 years)	Men (*n* = 1,464)	258 (755)	706 (553)	91 (220)
	Women (*n* = 1,552)	181 (566)	550 (467)	184 (335)
Finland, 2017 (18–74 years)	Men (*n* = 780)	117 (89–145)^2^ 38 (28–48)^3^	493 (466–521)	81 (68–94)
	Women (*n* = 875)	62 (52–72)^2^ 24 (17–30)^3^	382 (357–406)	145 (122–167)
Iceland, 2010 (18–80 years)	Men (*n* = 632)	274 (358)	404 (432)	40 (114)
	Women (*n* = 680)	205 (317)	272 (279)	72 (151)
Norway, 2010 (18–70 years)	Men (*n* = 862)	282 (396)	591 (508)	108 (251)
	Women (*n* = 925)	202 (329)	454 (452)	238 (355)
Sweden, 2010 (18–80 years)	Men (*n* = 792)	132 (234)	370 (290)	88 (161)
	Women (*n* = 1,005)	95 (157)	311 (256)	145 (218)
Estonia, 2014 (18–74 years)	Men (*n* = 907)	82 (159)	261 (199)	212 (218)
	Women (*n* = 1,806)	40 (137)	252 (252)	240 (322)
Latvia, 2020 (19–64 years)	Men (*n* = 470)	128	303	210
	Women (*n* = 541)	61	298	179
Lithuania, 2019 (19–75 years)	Men (*n* = 1,348)	74 (198)	348 (258)^1^	
	Women (*n* = 1,562)	36 (102)	356 (238)	

Data are provided as mean and SD or 95% Confidence Interval (Finland). Data are retrieved from the respective country’s national dietary survey in adults.

1 Coffee, tea, and cocoa (in liquid form);

2 Sugar-sweetened beverages;

3 Artificially sweetened beverages.

The intake of added sugars from SSBs in Nordic and Baltic countries is shown in Table 2. Data were retrieved from the EFSA report published in 2022 (18), with data from Denmark, Estonia, Finland, Latvia, and Sweden. Although these were the most recent data presented collectively, not all data might be up to date as countries regularly conduct national dietary surveys. The added sugars in SSBs contribute 1 to 7 percentage of total energy intake (E%) among children, adolescents, and adults. Among the group with very high intake of added sugars (i.e. the 95th percentile), the added sugars in SSBs contribute with 2 (among children in Latvia) to 24 E% (among adolescents in Denmark) (23).

**Table 2:** Intake of added sugars from sugar-sweetened beverages (expressed as E%) in different age groups and countries

Age group	Children		Adolescents (10–14 years)		Adults	
Country	Mean	High added sugar consumers	Mean	High added sugar consumers	Mean	High added sugar consumers
Denmark	6	20	7	24	5	22
Estonia	1	8	2	19	1	4
Finland	1	3	2	20	1	4
Latvia	0	2	1	14	1	4
Sweden	4	15	4	13	2	9

Average intake and among the group with very high consumption of added sugars (i.e. 95th percentile). Data were obtained from the EFSA report’s supplementary information (Annex D), which have used data from respective country´s national dietary surveys (24). These were the most recent data presented collectively, not all data might be up to date as countries regularly conduct national dietary surveys.

## Effect of beverages on health outcomes

### Coffee

#### Cancer

According to the summary from the WCRF, there is strong evidence from observational studies that consuming coffee probably decreases the risk of liver cancer and endometrial cancer. In addition, the evidence suggesting that the consumption of coffee decreases the risk of cancers of the mouth, pharynx, and larynx, and of skin cancer (basal cell cancer in men and women; malignant melanoma in women) is limited (17).

#### Cardiovascular diseases

An umbrella review from 2017 summarized the evidence on coffee and multiple health outcomes from meta-analyses published before July 2017 (24). One meta-analysis of 31 prospective cohort studies reported a benefit from drinking three cups of coffee per day regarding all-cause cardiovascular, coronary heart disease, and stroke mortality. The association was non-linear, with the beneficial effect of drinking more than 3 cups/day being less pronounced (25). Another meta-analysis, which included 28 prospective cohort studies, reported a reduction of risk of cardiovascular mortality for each additional coffee cup consumed per day (26), and one meta-analysis of two studies found an inverse association between coffee consumption and myocardial infarction mortality (27). Coffee consumption was also found to be inversely associated in a non-linear fashion with overall cardiovascular disease (CVD), coronary heart disease, and stroke incidence, as reported by another meta-analysis that summarized the results of 36 cohort studies. The largest benefit was found in those who drank between 3 and 5 cups/day, and there was no significant difference in the risk profile between men and women (28). Two separate meta-analyses found no significant association between coffee consumption and the risk of venous thromboembolism (two cohort and one case-control study) and atrial fibrillation (two cohort studies) (29, 30). One meta-analysis of five cohort studies reported an inverse association between drinking four cups of coffee per day and the risk of heart failure (31). A meta-analysis of 10 cohort studies published after 2017 found that coffee consumption was inversely and linearly associated with cardiovascular and coronary heart disease mortality in patients with type 2 diabetes. In the same population, drinking four cups of coffee per day was also found to be inversely associated with coronary heart disease and overall CVD risk (32).

#### Hypertension

There are currently conflicting results regarding coffee consumption and risk of hypertension. Three meta-analyses of prospective cohort studies found that high coffee consumption was not associated with increased blood pressure and in fact report a non-linear inverse dose-response relationship between coffee consumption and the risk of hypertension (33–35). On the other hand, a meta-analysis that included 11 randomized controlled trials (RCTs) with combined 470 study subjects (mean age 27 years), with a study duration ranging from 30 min to 4 weeks, found that caffeinated beverages (coffee and other caffeinated beverages) were associated with an overall short-term (within 4 weeks) blood pressure elevation of 3.04/2.45 mmHg. This association was stronger in adolescent population compared to adults (36).

#### Dyslipidemia

A meta-analysis that included 12 RCTs found that the consumption of unfiltered coffee had an unfavorable effect on serum low-density lipoprotein (LDL) and triglyceride levels. At the same time, the consumption of boiled coffee was associated with lower serum levels of high-density lipoproteins (HDLs). This effect was not observed with the consumption of filtered coffee (37). In the most recent meta-analysis of 12 RCTs, coffee consumption was found to be associated with significantly increased total cholesterol, triglycerides, and LDL levels but had no significant effect on HDL cholesterol levels. This study also found a non-linear relationship between coffee consumption and the increase in total cholesterol, LDL cholesterol, and triglyceride levels (38).

#### Diabetes

Four meta-analyses of prospective cohort studies were found on the association between coffee consumption and the risk of type 2 diabetes (39–42). All four studies found an inverse association; caffeinated and non-caffeinated coffee types having a similar risk profile and a dose-response relationship were also found in three meta-analyses (39, 40, 42). One meta-analysis reported that the inverse association was more pronounced in women, and that the strongest effect was observed in non-smokers and in those with a body mass index (BMI) below 25 kg/m^2^ (40).

#### Liver outcomes

One meta-analysis of five cohort studies found that regular coffee drinkers have lower risk of non-alcoholic fatty liver disease (NAFLD) compared to non-drinkers (43). Two meta-analyses (nine cohort and seven case-control studies; five cohort and four case-control studies) reported that coffee consumption decreased the risk of liver cirrhosis (44, 45). On the other hand, an umbrella review of four meta-analysis on coffee consumption and NAFLD found no clear benefits of coffee drinking on risk of NAFLD in general population after pooling the results from nine studies. At the same time, among patients with NAFLD, coffee consumption was found to decrease the incidence of liver fibrosis (46).

#### Obesity

A single meta-analysis from 2019, which included 12 studies, found that coffee intake was modestly associated with a decrease in fat mass, and that the effect was more pronounced in men (47). Out of 12 included studies, 11 were cross-sectional studies, and only one was a prospective cohort study that evaluated the effect of 15 years of coffee consumption on the five components of the metabolic syndrome.

#### Neurological disorders

The results from five meta-analysis that all combined results from several types of observational studies (cohort, case-control, and cross-sectional) suggest that coffee consumption was associated with decreased risk of Parkinson’s disease (48–52). Two meta-analyses, each including cohort, case-control, and cross-sectional studies, found an inverse association between coffee intake and the risk of depression (53, 54), and an inverse association between coffee intake and the risk of Alzheimer’s disease was found in a meta-analysis including 11 prospective studies (55).

#### Pregnancy outcomes

The consumption of caffeine and coffee during pregnancy was found to be associated with increased risk of pregnancy loss, as reported by two meta-analyses of observational studies (56, 57). Similarly, in two meta-analyses (15 cohort studies, and nine cohort and four case-control studies), high caffeine intake during pregnancy was associated with a higher risk of low birth weight with a linear dose-response relationship (58, 59). Finally, a meta-analysis of 15 cohort and seven case-control studies showed that caffeine intake during pregnancy increased the risk of preterm birth, and that the association was observed in both cohort and case-control studies (60). It is worth noting that the assessment of caffeine intake in the mentioned meta-analyses differed based on the included studies. While some studies included multiple sources of caffeine, for example, coffee, tea, and cola drinks, other studies estimated caffeine intake based on coffee consumption alone.

#### Mendelian randomization studies using genetic variation as a proxy for coffee consumption

Mendelian randomization studies can improve causal inference by using genetic variants as instrumental variables (proxies) for an exposure, and thereby reduce the influence of confounding and reverse causation. Several genetic variants strongly associated with coffee consumption have recently been identified (61). The present Mendelian randomization studies found no evidence in support of a causal association between coffee consumption and CVD risk (62–65), type 2 diabetes (66, 67), or hypertension (68). However, there is some support for unfavorable lipid profiles of high coffee consumption (69), and some support for a protective role in NAFLD risk (70, 71). In addition, there is some support for a detrimental role of coffee consumption in Alzheimer’s disease (72–74). The current Mendelian randomization studies found no support for causal effect on miscarriage, stillbirths, preterm birth or effect on gestational age (75), or pregnancy loss (76).

### Tea

#### Cancer

According to the summary from the WCRF, there is limited evidence from observational studies that suggest that tea consumption decreases the risk of bladder cancer. No recommendations have been made as more evidence is necessary to assess an anti-tumorigenic role of tea consumption in bladder cancer (17).

#### Cardiovascular diseases

Data from 39 prospective cohort studies suggest that daily tea consumption might be associated with lower risks of CVD mortality. The meta-analysis showed that daily consumption of black and green tea was associated with a 4% lower risk of CVD mortality, a 2% lower risk of CVD events, and a 4% lower risk of stroke (77). A meta-analysis that summarized the results from 18 prospective cohort studies found a 5% lower risk of CVD mortality for every additional cup of green tea per day (78). However, in a recent critical appraisal of the evidence on tea consumption and cardiovascular health and disease, it was concluded that the body of evidence to date is not of sufficient quality to make recommendations regarding an optimal level of tea intake that could be used to improve cardiovascular health (79).

#### Hypertension

A meta-analysis from 2014 included 13 RCTs with study duration varying between 3 weeks and 3 months with the aim to quantify the association between green tea intake and blood pressure. The authors found that both systolic and diastolic blood pressure decreased in green tea arms (weighted mean differences -1.98 mmHg and -1.92 mmHg, respectively) (80).

A more recent meta-analysis, which included five RCTs, found that the consumption of black or green tea led to a decrease in both systolic (weighted mean difference -4.81 mmHg) and diastolic blood pressures (weighted mean difference -1.98 mmHg). The authors observed that longer duration of tea intake was associated with a higher decrease in systolic and diastolic blood pressures (81).

#### Diabetes

Daily tea consumption of three or more cups of tea per day was associated with a lower type 2 diabetes risk in a meta-analysis of 12 cohort studies, with an inverse association being observed in women only (82). A meta-analysis from 2021, which included 14 RCTs, found that green tea had no significant effect on fasting plasma glucose, fasting insulin, and HbA1c in patients with type 2 diabetes (83).

#### Obesity

The results from a meta-analysis that included 26 RCTs in which the follow-up period ranged from 2 weeks to 5 months suggest that green tea supplementation led to a decrease in body weight (weighted mean difference –1.78 kg; 95% confidence interval [CI]: −2.80, −0.75) and BMI (−0.65 kg/m^2^; −1.04, −0.25) in individuals with obesity (84). The recommended daily dose of green tea in the included studies ranged from 99 to 20,000 mg and included both green tea in sachets and green tea extract administered via capsules.

#### Neurological disorders

The results from a meta-analysis that included three cohort and nine case-control studies suggested that tea consumption was associated with a reduced risk of developing Parkinson’s disease. However, after stratifying the analysis according to the study design, no significant association remained in either cohort or case-control studies (85). A linear, inverse association between green tea and the risk of cognitive disorders was found in a meta-analysis of 17 observational studies. No significant association was found with black tea (86).

### Sugar-sweetened beverages

EFSA has published a scientific opinion on the tolerable upper intake level for dietary sugars, that is, the maximum level of chronic daily intake of sugars judged to be unlikely to pose a risk of adverse health effects to humans (18). They performed systematic reviews of the relationship between intake of sugars (including SSB) and chronic metabolic diseases, pregnancy-related endpoints, and dental caries. The search was conducted in July 2018 and updated in August 2020 (metabolic diseases) or October 2020 (dental caries). The studies included in the body of evidence allow investigation of SSB in relation to the outcomes.

#### Obesity

In the EFSA report, six RCTs, lasting between 12 and 72 weeks, assessed the consumption of SSBs versus sugar-free alternatives. Five of the RCTs were conducted in individuals with overweight or obesity, and two were in children and adolescents. Body weight was higher in the SSB group than the sugar-free alternative in all studies, and statistically significant in two studies. All 10 prospective cohort studies reported positive associations between the intake of SSBs and incidence of obesity and/or abdominal obesity. The association was statistically significant in six out of the seven studies, which did not keep total energy intake constant in the analyses, and in one out of three studies which kept energy intake constant. Most of these studies were conducted in children or adolescents. All of the 13 prospective cohorts (six of these in children) investigating the relationship between change in SSBs intake and measures of body weight or BMI reported positive associations, and these were statistically significant in eight studies (18).

From the US Dietary Guidelines 2020 Advisory Committee, one of the systematic reviews with the following research question could be used: ‘What is the relationship between beverage consumption and growth, size, body composition, and risk of overweight and obesity?’. From this systematic review, moderate evidence indicates that higher SSB intake is associated with greater weight gain and obesity in children, while limited evidence suggests that high SSB intake is associated with greater weight gain and obesity in adults (19).

#### Type 2 diabetes

In the EFSA report, a positive relationship between the intake of SSBs and the incidence of type 2 diabetes was observed in 14 out of 15 prospective cohort studies. The association was attenuated when BMI was included in the model in many of the studies, suggesting that the relationship may be in part mediated by BMI. The results were mixed from the seven RCTs that were conducted with high versus low intake of SSBs on fasting glucose with an overall non-significant overall difference in effect (18).

#### NAFLD

Liver fat was significantly higher in the high versus low SSB arms in the three available RCTs (all in overweight or obese individuals). Beverages were consumed ad libitum or under neutral energy balance. Only one prospective cohort study was available, observing a significant positive linear dose-response relationship between the intake of SSBs and changes in visceral adipose tissue (18).

#### Dyslipidemia

The results of the seven available RCTs comparing high versus low SSB doses, or SSB to a sugar-free alternative, were mixed for all blood lipids. Most RCTs were conducted ad libitum, among individuals with overweight or obesity, and with differences in sugar from beverages between 8 and 22 E%. Five prospective cohort studies, four of which were in adults and one in children and adolescents, investigated the relationship between the intake of SSBs and the incidence of high triglycerides and low HDL-cholesterol. Three also investigated the relationship with the incidence of high LDL-cholesterol. Most of the prospective cohort studies reported positive and non-significant relationships between the intake of SSBs and the incidence of high triglycerides, low HDL-cholesterol, and high LDL-cholesterol (18).

#### Hypertension

There is evidence from prospective cohort studies for a positive and causal relationship between the intake of SSBs and the risk of hypertension. All cohort studies report a positive association between the intake of SSBs and the incidence of hypertension, and the associations were significant in four of the seven cohorts. The evidence from the four available RCTs with individuals with overweight or obesity showed mixed results (18).

#### Cardiovascular disease

No RCTs on CVD endpoints were available. Four out of five prospective cohort studies that investigated the relationship between the intake of SSBs and CVD incidence or CVD mortality reported a positive association. In the study that did not show an association, total energy intake was kept constant in the analysis. The pooled hazard ratio for the highest versus lowest intake category was 1.15 (95% CI: 1.03–1.29). Among the six studies reporting on coronary heart disease incidence or mortality, three showed a positive (non-significant) association, and three showed an association close to null. The pooled hazard ratio for the highest versus lowest intake category was 1.08 (95% CI: 1.00–1.18). The results for SSBs and stroke varied, and the pooled hazard ratio for the highest versus lowest category was 1.07 (95% CI: 0.96–1.19) (19).

### Low and No-Calorie Sweetened Beverages

As a basis to the US Dietary Guidelines 2020, a systematic review was conducted, which examined the relationship between LNCSB and outcomes related to body composition, and risk of overweight and obesity (19). In studies examining LNCSB intake in children (based on 17 prospective cohort studies), most studies (about 75%) reported no association with the overweight and obesity outcomes, and the remaining studies showed either increased or decreased risk of obesity with high LNCSB intake. In studies examining LNCSB intake in adults (based on six articles from RCTs, with 12 weeks to 1 year of duration, and 14 articles from prospective cohort studies, with 2 to 20 years of follow-up), most studies (72%) reported a significant effect or association between LNCSB intake and reduced body weight. For example, one well-designed RCT and two large prospective cohort studies reported an association between LNCSB and reduced body weight. In conclusion, limited evidence suggests no association between LNCSB consumption and body weight in children, and limited evidence suggests that LNCSB consumption is associated with reduced body weight in adults (19).

In a systematic review from the WHO from 2022, using data from RCTs, the consumption of non-sugar sweeteners was found to result in a small reduction in body weight in adults, without significant effects on other cardiometabolic risk markers, including fasting glucose, insulin, blood lipids, and blood pressure. The effect on body weight was more pronounced when non-sugar sweeteners were compared to sugar, and likely mediated through the effect of reduced energy intake. In contrast, using data from observational studies, higher intake of non-sugar sweeteners was associated with increased risk of type 2 diabetes, CVD, and mortality. Results for pregnant women suggest that higher intake of non-sugar sweeteners is associated with increased risk of preterm birth (low certainty evidence) and possible adiposity in offspring (very low certainty evidence) (20).

We also conducted a scoping review of systematic reviews on artificially sweetened beverages and health outcomes. We found several recently conducted systematic reviews of prospective cohort studies on LNCSB beverages and mortality (87–92), type 2 diabetes (87, 92–94), CVD (87, 91), or hypertension (92, 95, 96). With each additional serving of artificially sweetened beverages, the risk increased by 13% for type 2 diabetes, 8% for CVD, and 7% for all-cause mortality (87). Another systematic review found an 8% increased risk of hypertension for each serving (92). However, many of these associations were found to be non-linear. For example, there seemed to be a J-shaped association for all-cause and CVD mortality with increased risk above 1.5 servings/day (90). Higher intakes were also associated with increased risk of obesity in prospective cohort studies (92). A favorable effect on body weight is suggested (evidence from two cohort studies and three RCTs) when SSBs are replaced by water, coffee, tea, or LNCSB (97).

The conflicting results of health effect of LNCSB from the RCTs and prospective cohort studies are not easy to explain. However, reverse causation is a possible explanation for observed associations in observational studies. That is, individuals at high risk of disease and with adiposity choose LNCSB as a mean to reduce their energy intake. This may introduce spurious associations between LNCSB and the risk of development of diseases (20).

#### Mechanisms

Several substances found in coffee may explain its health effects. It has been hypothesized that chlorogenic acid from coffee could have a favorable effect on type 2 diabetes risk by reducing glucose levels in the blood and increasing insulin sensitivity (98). Some studies proposed that the reduced risk of cardiovascular deaths among heavy coffee consumers is due to a decreased resting heart rate (99, 100). On the other hand, coffee diterpenes can increase serum cholesterol and might cause extracellular accumulation of LDLs (101, 102). This effect is more pronounced in the coffee types that are rich in diterpenes, such as boiled coffee. Filtered coffee have lower levels of diterpenes, as the paper filter blocks the passage of fine particles into the brew, and diterpenes are retained by the spent coffee (103, 104). Caffeine was found to improve both physical and cognitive performances, and to have antioxidant properties (105–107). Adverse effects of caffeine are either consequences of excessive amount of the substance consumed or withdrawal from caffeine. These effects usually include prolonged headache, heart rhythm irregularities, insomnia, and increase in anxiety and jitteriness (108,109). Green tea is rich in flavonoids such as catechin. It was previously suggested that catechins could regulate blood pressure by protecting vascular endothelial cells and improving vascular integrity (110).

Excess energy intake leading to positive energy balance and weight gain is the main mechanism by which the intake of SSBs can contribute to the risk of chronic metabolic diseases. Calories from liquid sources have been found to have lower effect on satiety compared to solid foods (111); however, more research is needed. The small reduction in body weight observed in intervention groups consuming LNCSB compared to intervention group consuming SSB is likely mediated through the effect of reduced energy intake. In a systematic review of observational studies, the substitution of LNCSB for SSB was associated with lower incidence of obesity, coronary heart disease, and mortality (112).

## Food-based dietary guidelines

### Coffee and tea

The body of evidence available suggests that moderate coffee and tea consumption do not have long-term adverse effects on health. The acute adverse effects such as prolonged headache, heart rhythm irregularities, insomnia, and increase in anxiety and jitteriness are most common results of a high caffeine intake in a short time interval or are the side effects of caffeine withdrawal. The observed long-term favorable effects of coffee consumption are related to reduced risk of endometrial and liver cancer sites, type 2 diabetes, and cardiovascular deaths. On the other hand, results from RCTs suggest that coffee brews that are rich in diterpenes, such as boiled coffee, increase the levels of serum cholesterol. Pregnant women should not exceed the recommended daily dose of caffeine intake. There is some evidence on beneficial effect of black and green tea on blood pressure.

The evidence for beneficial effects of coffee consumption on certain health outcomes comes from observational studies. As most observational studies have a limited potential for causal inference due to inherited biases such as confounding and information bias, it is difficult to claim that the observed associations between coffee consumption and health outcomes are causal. The evidence from Mendelian randomization studies that improve causal inference does not, for example, support the causal effect on coffee consumption and CVD risk. However, these studies cannot assess whether there is a U-shaped association. More studies are needed on coffee brews that have become popular in the last two decades, such as macchiato, espresso, cappuccino, or caffe latte. Future observational studies on coffee/tea consumption and health outcomes should incorporate some newly developed causal models that allow drawing causal inferences from observational studies, such as the use of directed acyclic graphs, propensity score matching, and emulating a target trial using observational data.

#### SSB

According to the EFSA report, positive and causal relationships were identified between the intake of SSBs and the risk of obesity, type 2 diabetes, hypertension, and CVD (high certainty), NAFLD and dyslipidemia (low certainty) based on data from RCTs and prospective cohort studies. The dose-response relationships between the intake of SSB and the incidence of type 2 diabetes, hypertension, and CVDs (using data from prospective cohort studies) were positive and linear. Although the overall evidence from prospective cohorts on SSBs supports a positive and causal relationship between SSBs and the risk of chronic metabolic diseases, this was not the case for added and free sugars from all sources. Importantly, SSBs were analyzed not keeping total energy constant, which is different from the analyses for added and free sugars. The evidence for adverse effects on hard endpoint (such as diseases and death) is only available from observational studies. However, there are inherited biases in observational studies, such as confounding and information bias. Since the associations have been observed in many populations and adverse effects on risk markers were found in RCTs, the certainty of a causal effect for SSB is strengthened. The evidence suggests that there is a strong need to limit the consumption of added sugar from beverages.

#### LNCSB

The consumption of LNCSB may result in a small reduction in body weight in adults, likely mediated through the effect of reduced energy intake compared to intervention group consuming sugar but has neutral effects on other cardiometabolic risk markers. However, evidence from observational studies on increased risk of cardiometabolic diseases among high LNCSB consumers raises concern regarding potential adverse long-term effects. The conflicting results from RCTs and observational studies may, however, be due to revere causation and should be explored further before recommendations for LNCSB consumption can be given. WHO suggests that non-sugar sweeteners should not be used as a means of achieving weight control or reducing the risk of non-communicable diseases (conditional recommendation).

Although the search strategy that was used was comprehensive, and that hand search of references of relevant articles was also performed, there is still a possibility that some relevant studies were missed.

## Conflict of interest and funding

The authors have not received any funding or benefits from industry to conduct this study, and report no conflicts of interests. The authors received a small reimbursement from the Norwegian Directorate of Health for work linked to this article.
